# Self-efficacy and self-care-related outcomes following Alexander Technique lessons for people with chronic neck pain in the ATLAS randomised, controlled trial^☆^

**DOI:** 10.1016/j.eujim.2017.11.006

**Published:** 2018-01

**Authors:** Julia Woodman, Kathleen Ballard, Catherine Hewitt, Hugh MacPherson

**Affiliations:** a Society of Teachers of the Alexander Technique, Grove Business Centre, Unit W48, 560-568 High Road, London, N17 9TA, UK; b Department of Health Sciences, University of York, York, YO10 5DD, UK

**Keywords:** NPQ, Northwick Park Neck Pain Questionnaire, SF-12, short-form quality of life survey, Alexander Technique, Self-efficacy, Self-care, Neck pain, Chronic pain, Musculoskeletal, Randomised controlled trial

## Abstract

**Introduction:**

ATLAS was a pragmatic randomised (1:1:1 ratio), controlled trial recruiting patients with chronic neck pain (N = 517) and evaluating one-to-one Alexander Technique lessons, or acupuncture, each plus usual care, compared with usual care alone. The primary outcome (12-month Northwick Park Neck Pain Questionnaire [NPQ]) demonstrated significant and clinically meaningful reductions in neck pain and associated disability for both interventions compared with usual care alone. Here we describe pre-specified, self-efficacy and other self-care-related outcomes for the Alexander group compared with usual care.

**Methods:**

Participants reported on 11 self-efficacy/self-care-related outcome measures at 6 and 12 months. Linear or logistic regression models evaluated changes in parameters and impact on NPQ. Alexander teachers reported on lesson content.

**Results:**

Lesson content reflected standard UK practice. The Alexander group (n = 172) reported significantly greater improvements, compared with usual care alone (n = 172), in most of the self-efficacy/self-care measures (9/11 measures at 6 months, and 8/11 at 12 months), including the ability to reduce pain in daily life. At 6 months, 81% (106/131) of Alexander participants reported significant improvement in the way they lived and cared for themselves (versus 23% for usual care), increasing to 87% (117/135) at 12 months (usual care: 25%). NPQ scores at both 6 and 12 months were related to improvement in self-efficacy and ability to reduce pain during daily life.

**Conclusions:**

Alexander Technique lessons led to long-term improvements in the way participants lived their daily lives and managed their neck pain. Alexander lessons promote self-efficacy and self-care, with consequent reductions in chronic neck pain.

## 1 Introduction

Neck and back pain together now represent the leading cause of disability in all high income countries, and globally for the 25–64 year age group [1]. Chronic neck pain is regarded as often complex in origin and nature and particularly difficult to manage [2]. Furthermore, the challenge of chronic neck pain is likely to grow due to increasing computer and mobile technology use, with recognised consequences such as ‘text neck' [[3], [4], [5]].

One approach to the solution of this growing problem that warrants investigation, is to explore ways of encouraging better self-efficacy and self-care. In this research, self-efficacy is defined as confidence in one’s ability to execute a behaviour to produce a desired outcome [[6], [7]]. We define self-care broadly as a certain positive attitude and form of attention towards the self, in respect of any necessary function that is under individual conscious control and is self-initiated [[8], [9]]. Greater self-efficacy and self-care could enable individuals to recognise and reduce some of the underlying causes of musculoskeletal pain, such as mal-coordinated postural and movement habits, excessive muscular tension, and associated psychological distress [[3], [10], [11], [12]]. The Alexander Technique is an effective long established but often under-utilised way of bringing about such constructive self-change. It is an embodied reflective practice that enables individuals to improve the way they go about their daily activities, through increased awareness, intentional inhibition of unwanted reaction and unnecessary action, and with more effective direction of thought; all leading to improved overall muscle tone and postural support with less stiffness [[13], [14], [15], [16]]. The Technique is usually taught in one-to-one lessons, using integrated spoken and hands-on guidance [[17], [18]]. Such lessons have led to diverse health and performance-related benefits [[19], [20]]. Training in the Alexander Technique has been shown to increase dynamic postural muscle tone [21], and improve movement coordination and balance [[22], [23], [24]]. These movement and balance changes are thought to result from the altered postural tone [25]. Research studies, often using qualitative methods, have reported improvements in psychological well-being, mood and confidence, as well as reduction in performance-related anxiety following one-to-one Alexander lessons [[20], [26], [27], [28]].

The ATLAS (Alexander Technique Lessons or Acupuncture Sessions) trial is the second large randomised controlled study to evaluate the effectiveness of Alexander lessons in a chronic musculoskeletal pain population. The earlier ATEAM trial demonstrated that, compared with usual care alone, one-to-one Alexander lessons led to significant long-term reduction in chronic or recurrent back pain and associated disability, [18]. ATLAS compared usual care alone with either Alexander lessons or acupuncture (both plus usual care) for primary care patients with chronic (median 6 years) non-specific neck pain [[16], [29]]. The ATLAS trial clinical findings have already been reported, with the primary outcome of the Northwick Park Neck Pain Questionnaire demonstrating statistically significant and clinically meaningful reductions in pain and associated disability for both Alexander lessons and for acupuncture sessions compared with usual care alone, with the benefit maintained to at least 12 months [16]. The trial design encompassed a range of additional participant-reported outcomes that were pre-specified in the protocol, mostly relating to self-efficacy and the ability to improve self-care [29]. In addition to the outcome data collected from participants, data were also collected from the practitioners regarding delivery of Alexander lessons and acupuncture. Findings for the acupuncture group have been published separately [[16], [30]]. Here we report the results for the self-efficacy and other self-care-related outcomes in the Alexander group.

The main aims of the current analysis are: to evaluate the extent of change in self-efficacy and self-care ability during and following a series of one-to-one lessons in the Alexander Technique; to compare the extent of any such changes with those in the group receiving usual care alone; and to identify any relationships between such changes and the long-term clinical outcome already reported in this chronic neck pain population. The ATLAS trial was not designed for direct comparison of Alexander lessons and acupuncture; however, based on descriptive analyses, we report similarities and differences between the outcomes for the two interventions [30], as a means of gaining insight into their distinctive natures. An additional objective is to report on the content of the Alexander lessons delivered in the trial.

## 2 Methods

The design and methodology for the ATLAS trial (Current Controlled Trials, ISRCTN15186354) have been described in full elsewhere and are briefly summarised here [[16], [29]].

### 2.1 Study design and participants

ATLAS (Alexander Technique Lessons or Acupuncture Sessions) was a pragmatic, three-arm randomised controlled trial that recruited people who had consulted their primary care practitioner (GP) for chronic, non-specific neck pain. GP surgery databases were searched for potential participants who were invited to complete a baseline questionnaire, screened later for eligibility. Inclusion criteria were: age ≥18 years, neck pain duration ≥3 months, and a Northwick Park neck pain and associated disability Questionnaire (NPQ) score of ≥28% [[31], [32]]. Exclusion criteria included serious underlying pathology. Eligible participants were randomised in a 1:1:1 ratio to Alexander lessons plus usual care, acupuncture plus usual care, or usual care alone. In total, 517 patients were recruited and randomised between March 2012 and April 2013.

Written informed consent was obtained from all participants and ethical approval from Leeds West Research Ethics Committee (REC ref 11/YH/0402).

### 2.2 Alexander Technique teachers

All participating Alexander teachers were members of the Society of Teachers of the Alexander Technique (STAT) with at least 3 years' teaching experience and a declared commitment to their continuing professional development. Teaching methods involved verbal and hands-on guidance in line with usual practice and UK-based National Occupational Standards Skills-for-Health guidelines [33].

### 2.3 Interventions

Participants randomised to the Alexander group were offered a total of 20 one-to-one lessons, each 30-minutes’ duration (600 minutes total) plus continued usual medical care. Lessons were typically weekly, with the option of being twice-weekly initially and later fortnightly, with the intention of completion within 5 months. Participants randomised to the acupuncture group were offered an equivalent intervention duration of traditional Chinese acupuncture plus continued usual medical care. All participants received usual care which consisted of treatment routinely provided to primary care patients (both general and neck pain-specific), such as prescribed medications and visits to other healthcare professionals, for example physiotherapists.

### 2.4 Participant-reported outcomes

The primary outcome measure for the trial was the Northwick Park Neck Pain and associated Disability Questionnaire (NPQ), and these findings, together with secondary clinical outcome measures, have been reported elsewhere [16]. Additional outcome measures were included in the participant questionnaires that were completed at baseline, 6 and 12 months. Self-efficacy was determined by the five-question pain management sub-scale of the Chronic Pain Self-Efficacy Scale. In line with previous studies, we used the validated modified version in which the original 0−10 scale is replaced with 0−8 and ‘certain’ replaced with ‘confident’ [[6], [7], [34]]. The questions in this scale, scored 0 (totally unconfident) to 8 (totally confident), were ‘How confident are you that you can: i) decrease your pain quite a bit?; ii) continue most of your daily activities?; iii) keep pain from interfering with your sleep?; iv) make a small-to-moderate reduction in your pain by using methods other than taking extra medications?; v) make a large reduction in your pain by using methods other than taking extra medications?’. The four-item version of the Perceived Stress Scale was also used, and asked the following questions, scored 0 (never) to 4 (very often): ‘In the last month, how often have you i) felt that you were unable to control the important things in your life?; ii) felt confident about your ability to handle your personal problems?; iii) felt that things were going your way?; iv) felt difficulties were piling up so high that you could not overcome them?’ [[35], [36]]. Other questions included in the participant questionnaire at 6 and 12 months were: 1. ‘Can you use/apply the things you have learned from the care in everyday life situations to reduce pain?’, a question modified from one that was used to assess self-management in a previous neck pain trial (‘reduce’ replacing ‘cope with’) [37]; 2. ‘During the care you received in the last 6/12 months, did you learn to improve the way you live and care for yourself?’; 3. ‘To what extent are you able to put into practice the advice or teaching you received?’; 4. ‘To what extent are the changes you have been making helpful to you?’; 5. ‘Did you make any changes related to a) diet, b) exercise, c) relaxation, d) rest, e) work'?

### 2.5 Practitioner-reported data

Following each lesson, the Alexander teachers recorded lesson content in a participant-specific log book that listed the basic components of lessons in terms of Alexander principles to explore, and practical activities that might be engaged in to help people discover how to apply the principles and improve their skill and understanding. Teachers recorded additional information in the log book when the participant had finished attending lessons.

### 2.6 Statistical analyses

Participants were analysed in the groups to which they were randomised, regardless of intervention adherence. Analyses were conducted in Stata version 13 (StataCorp. 2013. *Stata Statistical Software: Release 13*. College Station, TX: StataCorp LP). All analyses of participant-reported data were pre-specified in the published protocol [29] (the trial was registered prior to patient recruitment beginning), and in a statistical analysis plan (which was finalised prior to data analysis). Analyses of data from the teacher log books were pre-specified prior to data lock. Assumptions were checked for all analyses and no transformations or adjustments were required.

Descriptive data were reported as means and standard deviations, or median, minimum and maximum for continuous variables, and counts and percentages for categorical variables.

Ability to make improvements in living/self-care and any changes in diet, exercise, relaxation, rest and work at 6 and 12 months were analysed individually by logistic regression. Self-efficacy, Perceived stress, ability to use what has been learnt, extent to which advice or teaching were put into practice, and extent to which changes were helpful−all at 6 and 12 months, were analysed individually by linear regression. To explore the impact of participant-reported variables measured during the intervention period and changes in NPQ outcomes at 6 and 12 months, either linear or logistic regression was utilised. NPQ outcomes were analysed individually at each time point and included the participant-reported variables (or changes in these variables from baseline) as fixed effect covariates in the model.

Analyses were undertaken to explore if baseline factors might predict outcome independent of any intervention effect, with the baseline factor of interest included individually as a covariate in a linear regression model using NPQ score at 12 months as the outcome and adjusting for the same covariates as in the primary analysis [16]. Other analyses explored whether intervention effects varied among the levels of these baseline factors by extending this model to also include an interaction term between the potential moderator and intervention in the regression model. Estimates (including odds ratios for binary data) and 95% confidence intervals were presented (where appropriate) for each model.

All regression models adjusted for baseline NPQ, age, neck pain duration, gender and city as fixed effects and GP practice as a random effect using robust standard errors (Stata *regress* command with *cluster* option).

The teacher log book data were analysed using linear regression. These analyses were within-group only, since different data were collected for the Alexander lesson and usual care alone groups.

## 3 Results

### 3.1 Baseline characteristics

The baseline characteristics of the 344 participants were similar across the Alexander Technique lesson and usual care alone groups (Table 1). The study population included more women than men (69% versus 31%, respectively), was predominantly white (89%), with mean age of 54 years (SD 14), and a mean age of leaving full-time education of 18 years. More than half of participants (61%) were currently in paid employment, although 8% had reduced their hours and 7% had stopped working altogether because of their neck pain (Table 1).

**Table 1:** Baseline demographics.

	Alexander Technique lessons (N = 172)	Usual care alone (N = 172)	Overall (N = 344)
Mean age, years (SD)	53.62 (14.59)	53.85 (12.95)	53.74 (13.77)
Gender: Female, n (%)	120 (69.8)	118 (68.6)	238 (69.2)

Ethnicity, n (%)			
White-British	151 (89.4)	152 (88.9)	303 (89.1)
Indian	4 (2.4)	3 (1.8)	7 (2.1)
Bangladeshi	0 (0.0)	0 (0.0)	0 (0.0)
Pakistani	4 (2.4)	2 (1.2)	6 (1.8)
Chinese	1 (0.6)	1 (0.6)	2 (0.6)
Afro-Caribbean	1 (0.6)	0 (0.0)	1 (0.3)
Other	8 (4.7)	13 (7.6)	21 (6.2)

Mean age left full-time education, years (SD)	18.20 (6.13)	18.58 (5.98)	18.39 (6.05)

Outcome measures at baseline, mean (SD)			
NPQ % score	39.38 (11.91)	40.46 (11.60)	39.92 (11.75)
SF-12 Physical Component score	39.87 (9.75)	40.98 (9.49)	40.42 (9.62)
SF-12 Mental Component score	45.63 (12.22)	46.59 (10.87)	46.11 (11.56)
Perceived Stress Scale score	6.46 (2.96)	6.15 (3.36)	6.31 (3.17)
Chronic Pain Self-efficacy Scale	4.18 (1.53)	4.17 (1.54)	4.17 (1.53)

Employment status, n (%)			
Currently in paid employment	100 (59.2)	106 (62.0)	206 (60.6)
Reduced hours due to neck pain	11 (9.1)	8 (6.5)	19 (7.8)
Stopped working due to neck pain	10 (6.2)	12 (7.4)	22 (6.8)

SD: standard deviation; NPQ: Northwick Park Neck Pain Questionnaire score; SF-12: short-form quality of life survey (6- and 12-month outcomes for SF-12 have been reported previously [16]; There were 3 missing responses for ethnicity in the Alexander group and 1 for the usual care group; 8 missing responses for age left full-time education in each group; 3 missing responses in each group for the SF-12; 1 missing response on the Perceived Stress Scale and Pain Self-Efficacy Scale in the Alexander group; 3 missing responses for in paid employment in the Alexander group and 1 in the usual care group; 51 missing responses for reduction in hours in the Alexander group and 48 in the usual care group; 11 missing responses for stopping work due to neck pain in the Alexander group and 10 in the usual care group.

### 3.2 Intervention delivery and adherence

There was a wide range in the number of participants (1–21 participants, median: 8) taught by each of the 18 Alexander Technique teachers; 137 (40%) participants were located in Leeds, 123 (36%) in York, 47 (14%) in Manchester and 37 (11%) in Sheffield.

**Fig. 1:**
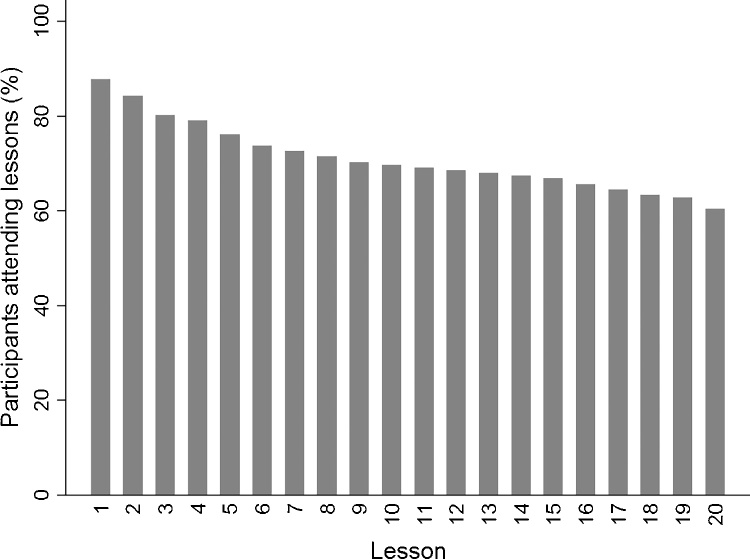
Intervention adherence: Percentage of participants attending Alexander Technique lessons over the 20 lessons offered.

Adapted with permission from MacPherson, H. et al. Alexander Technique lessons or acupuncture sessions for persons with chronic neck pain: A randomized trial. Annals of Internal Medicine 2015;163:653-662 (Appendix). Doi:10.7326/M15-0667. ©American College of Physicians.

The majority of participants in the Alexander Technique group (60%, 104/172) attended all 20 of the lessons offered. The remaining 40% attended a mean of 5.4 lessons (SD 6.3; range: 0 to 19), including 12% (21/172) who did not attend any. Overall, the mean number of lessons attended was 14.2 (SD 8.2; range 0 to 20). Dropout from the Alexander lesson group was most frequent in the period prior to and during the first few lessons; thereafter dropout was low and more evenly spread (Fig. 1). Where fewer than 20 lessons were attended (n = 68), discontinuation was initiated by: the participant (22%), by the teacher (13%), due to loss of contact (15%), due to crisis (6%), for other reasons (15%), or due to lessons never having begun (30%).

### 3.3 Participant-reported outcomes

Nine of the eleven outcomes differed significantly between the Alexander lesson and usual care alone groups at 6 months, and this difference was maintained at 12 months for eight of the outcomes (Table 2 ). Self-efficacy was significantly greater for the Alexander lesson group than for usual care at both 6 and 12 months. Furthermore, at both time-points, the Alexander group reported significantly greater ability to apply what had been learnt in the Alexander lessons to reduce pain in their daily lives, than was reported by the usual care group based on their experience of care received (Table 2). For the perceived stress score there was little difference between the Alexander and usual care groups at either 6 or 12 months.

**Table 2:** Participant-reported self-efficacy and self-care-related outcomes following Alexander Technique lessons compared with usual care alone at 6 and 12 months.

Outcome	6 months				12 months			
	Alexander Technique lessons Mean (SD; N)	Usual care alone Mean (SD; N)	Difference from usual care alone (95% CI)	p-value	Alexander Technique lessons Mean (SD; N)	Usual care alone Mean (SD; N)	Difference from usual care alone (95% CI)	p-value
Chronic Pain Self-efficacy Scale [scored 0: ‘totally unconfident' to 8:' totally confident'; higher scores indicate greater self efficacy]	5.05 (1.69; 134)	3.92 (1.52; 139)	1.09 (0.63 to 1.55)	<0.001	5.01 (1.78; 137)	4.14 (1.68; 139)	0.81 (0.37 to 1.24)	0.001
Perceived Stress Scale [0 to 16; lower scores indicate less stress]	5.54 (3.27; 136)	5.67 (3.23; 144)	-0.02 (-0.84 to 0.80)	0.97	5.63 (3.32; 139)	5.84 (3.48; 140)	-0.12 (-0.79 to 0.54)	0.70
'Can you use/apply things you learned from the care in everyday life situations, to reduce pain?' [0:'never' to 4: ‘everyday']	2.72 (1.10; 130)	1.45 (1.11; 125)	1.30 (0.94 to 1.66)	<0.001	2.59 (1.13; 140)	1.48 (1.09; 128)	1.11 (0.83 to 1.38)	<0.001
'To what extent are you able to put into practice the advice or teaching you received?' [0: ‘not at all' to 10: ‘completely]	6.59 (2.41; 116)	4.28 (3.20; 43)	2.20 (0.91 to 3.50)	0.002	6.21 (2.64; 126)	5.16 (3.26; 44)	1.01 (-0.20 to 2.21)	0.10
'To what extent are the changes you have been making helpful to you?' [0: ‘not at all' to 10: ‘completely]	6.62 (2.64; 117)	4.29 (2.98; 45)	2.23 (0.96 to 3.50)	0.001	6.28 (2.66; 123)	4.91 (2.95; 46)	1.21 (0.04 to 2.38)	0.04

Yes/No response questions	n/N answering ‘yes' (%)	n/N answering ‘yes' (%)	Odds ratio (95% CI)	p-value	n/N answering ‘yes' (%)	n/N answering ‘yes' (%)	Odds ratio (95% CI)	p-value
'Did you learn to improve the way you live and care for yourself?'	106/131 (80.9)	29/128 (22.7)	15.32 (8.79 to 26.68)	<0.001	117/135 (86.7)	31/123 (25.2)	23.98 (10.02 to 57.41)	<0.001
'Did you make any changes related to':	N = 143	N = 148			N = 145	N = 144		
Diet	12 (8.4)	8 (5.4)	1.82 (0.60 to 5.47)	0.29	20 (13.8)	13 (9.0)	1.69 (0.88 to 3.25)	0.12
Exercise	47 (32.9)	27 (18.2)	2.12 (1.12 to 3.98)	0.02	60 (41.4)	24 (16.7)	3.51 (1.99 to 6.21)	<0.001
Relaxation	93 (65.0)	13 (8.8)	19.76 (10.37 to 37.63)	<0.001	95 (65.5)	16 (11.1)	17.45 (8.92 to 34.14)	<0.001
Rest	74 (51.7)	8 (5.4)	20.31 (9.15 to 45.06)	<0.001	77 (53.1)	18 (12.5)	8.33 (4.64 to 14.99)	<0.001
Work	55 (38.5)	5 (3.4)	20.95 (8.68 to 50.54)	<0.001	55 (37.9)	12 (8.3)	6.98 (3.19 to 15.24)	<0.001

Adjustments were made for baseline NPQ score, duration of neck pain, age, gender and city as a fixed effect and GP practice as a random effect using robust standard errors; CI: confidence interval.

In the usual care group there was a low response rate to the two questions relating to putting into practice the advice or teaching received during the trial: 26% compared with the group overall response rate of 79% to the other questions, and contrasting with the Alexander group response rates of 70% and 80%, respectively (Table 2).

Some of the largest differences between the Alexander lesson and usual care group responses occurred in the ability of individuals to improve the way they lived and cared for themselves, with 81% of participants in the Alexander group reporting that they had learnt to improve this skill, and the figure rising to 87% at 12 months, while comparative values for usual care were 23% and 25%, respectively. Similarly, at both 6 and 12 months a much higher proportion of participants in the Alexander group than in the usual care group reported making changes relating to exercise, relaxation, rest and work, but not to diet (Table 2).

#### 3.3.1 Factors affecting clinical outcome

The greater self-efficacy, the level of ability to reduce pain by using/applying things learnt during the trial, the extent of ability to put into practice the advice or teaching received, the extent to which the changes were helpful, and the changes made to exercise and relaxation by the Alexander lessons group compared with the usual care group, were all found to be related to NPQ scores at both 6 and 12 months (Table 3 ). Learning to improve the way of living and caring for oneself was found to be related to NPQ score at 6 months but with weaker evidence by 12 months (Table 3).

**Table 3:** Impact of factors measured during the intervention period on Northwick Park Neck Pain and Disability Questionnaire (NPQ) scores at 6 and 12 months.

Parameter undergoing change following the intervention	Difference in NPQ % score between Alexander Technique lesson group and usual care alone group (95% CI)			
	6 months	p-value	12 months	p-value
Chronic Pain Self-Efficacy Scale	−3.34 (−4.03 to −2.64)	<0.001	−3.33 (−4.44 to −2.22)	<0.001
Perceived Stress Scale	0.78 (0.22 to 1.33)	0.008	0.98 (0.45 to 1.52)	0.001
Ability to use/apply things learned from the care in everyday life situations, to reduce pain	−2.72 (−4.14 to −1.29)	<0.001	−3.03 (−4.54 to −1.52)	<0.001
Extent able to put into practice the advice or teaching received	−1.09 (−2.03 to −0.15)	0.024	−1.82 (−2.57 to −1.08)	<0.001
Extent that changes made have been helpful	−1.79 (−2.45 to −1.12)	<0.001	−2.32 (−3.09 to −1.56)	<0.001

	Odds ratio (95% CI)	p-value	Odds ratio (95% CI)	p-value
Learnt to improve the way you live and care for yourself	−5.27 (−9.31 to −1.23)	0.012	−4.15 (−8.41 to 0.12)	0.057
Diet	−1.18 (−7.26 to 4.90)	0.685	1.00 (−5.91 to 7.90)	0.771
Exercise	−1.97 (−3.84 to −0.10)	0.039	−3.81 (−7.14 to −0.47)	0.027
Relaxation	−5.70 (−8.90 to −2.50)	0.001	−5.03 (−8.72 to −1.33)	0.009
Rest	−2.27 (−5.57 to 1.03)	0.171	−1.87 (−5.32 to 1.58)	0.278
Work	−3.70 (−7.70 to 0.30)	0.069	−2.28 (−5.55 to 0.98)	0.164

Data are from regression analysis with NPQ score as the outcome and intervention and characteristics included as covariates along with the same covariates as the primary analysis [16].

In separate analyses, baseline demographics (gender, age, ethnicity, employment or educational status), prior neck pain duration, and self-efficacy at baseline were not found to predict clinical outcome (NPQ score) at 12 months, or to moderate intervention effects (Supplementary Table 1).

#### 3.3.2 Outcome differences for trial interventions related to their key features

Some clear similarities and differences were seen between the self-care-related outcomes for the Alexander lesson and the acupuncture groups in this trial (acupuncture data reported in full elsewhere [30]). A descriptive comparison showed significant improvements, compared with usual care, during and following both interventions in the participants’ ability to improve their way of living and self-care, and in the changes they made relating to relaxation, rest and work – with a greater degree of such change in the Alexander group (Supplementary Table 2). Both groups reported an ability to apply what they had learnt from the intervention during everyday life situations and so reduce their pain. The difference from usual care for this parameter was statistically significant for the Alexander group, reflecting the fact that development of this ability is a key component of Alexander lessons. Participants in the acupuncture group alone made statistically significant dietary changes as a result of the intervention when compared with the usual care group (Supplementary Table 2), reflecting the fact that dietary advice is a key component of acupuncture practice but not of Alexander lessons.

### 3.4 Practitioner-reported data

Alexander Technique teachers reported being able to teach in line with their usual practice, with 93.8% (136/145) of the participants taught in this way. Log book data also revealed that lessons were generally organised around observation of, and reflection on, the manner of performing everyday activities such as sitting, standing, moving and lying resting in a particular way, while incorporating fundamental Alexander principles. Such activities provided a framework for the learning and practise of Alexander thinking and practical skills, and for the development of an understanding of the main principles; these had an integral role throughout each lesson (Supplementary Table 3).

## 4 Discussion

Individuals with chronic neck pain who attended Alexander Technique lessons reported improvements in a wide range of outcomes related to self-efficacy and self-care, with a significantly greater degree of positive change than occurred with usual care alone. These benefits were generally maintained over the 12 months of the study. Furthermore, many of these changes were found to be related to an improvement in clinical outcome, as assessed by the NPQ score at 12 months.

Strengths of this pragmatic study include the high overall adherence rate, with 60% of participants attending all 20 Alexander lessons and non-adherers generally being individuals who either never began their lessons (12%), or those who discontinued after one or two lessons. Furthermore, an overwhelming majority of participants (94%) were taught the Alexander Technique in line with their teachers' usual practice. This indicates that the trial design and conduct adequately reflected the ‘real world' setting and suggests that the results are likely to be transferrable to routine practice. The log book data completed by the Alexander teachers also revealed that the lesson content and delivery in the trial reflected current standard Alexander teaching practice across the UK, as shown by a recent large-scale national survey [17]. This survey did reveal some variation in teaching practice between the three participating professional organisations, but as the majority of Alexander teachers in the UK belong to the Society of Teachers of the Alexander Technique, as do all those who worked in this trial, the teaching in the ATLAS trial can be taken to be broadly representative of current overall UK teaching practice.

Limitations of the current analysis include low statistical power, resulting from the trial being powered only for comparison of groups for the main clinical outcome of NPQ score. In addition, only the participant-reported outcomes were pre-specified in the protocol; however, the practitioner-reported data analyses were specified in the statistical plan prior to data lock. A further limitation was the low response rate in the usual care group for the two outcome measures that related to putting into practice the advice or teaching received during the trial. Perhaps participants in this group did not perceive the relevance of these two questions? Their low response may have impacted the observed between-group differences for these two outcomes, particularly bearing in mind that the findings remained stable between 6 and 12 months for the Alexander group.

Although ATLAS was not designed, and therefore not powered, to be a comparative trial of Alexander Technique lessons and acupuncture sessions, the observed similarities and differences in self-efficacy and other self-care-related outcomes may nevertheless provide interesting insights into these two distinctive approaches to healthcare. The differences in findings between the two intervention groups regarding the nature and amount of benefit gained, demonstrate that benefit was not simply due to the additional (and equal) time and attention that participants received from the Alexander teachers and acupuncturists compared with the provision of usual medical care alone. Rather, the observed differences are most likely to have resulted from specific factors unique to each intervention. The participant-reported outcome measures employed can, therefore, be considered to provide meaningful data, even though some of them were devised specifically for the trial and were not validated measures. Previous trials have also demonstrated that long-term health benefits ensuing from Alexander Technique lessons are unlikely to result from non-specific effects of attention and touch [[18], [38]]. The predominantly self-care nature of the Alexander Technique and the distinctive teaching methodology employed were reflected in the outcomes for that group, such as the ability of individuals to reduce pain during the activities of daily life by applying the Alexander principles. Together with the other analyses for the acupuncture group [30], these findings accord with the view that acupuncture is predominantly therapeutic but with an important educational component primarily through lifestyle advice, while Alexander Technique lessons are primarily principle-based practical education and also have a therapeutic element.

To date there has been relatively little published research on the Alexander Technique and self-efficacy/self-care. However, participants in the ATEAM randomised trial who learnt the Technique reported that they were better able to manage their chronic back pain condition [39]. The same finding was reported by a service evaluation in a chronic pain clinic where Alexander lessons helped people manage self-perception of pain and reduce pain escalation, fear avoidance, and the effect of pain catastrophising [40]. These benefits enabled a reduction in reliance on medication, with more than half of the participants stopping or reducing their pain medication following lessons.

In the current analysis, participants who had a better clinical outcome in terms of neck pain and associated disability tended to be those who reported an increase in self-efficacy, and also those who reported being able to apply what they had learnt from their Alexander lessons during their daily lives to reduce pain. These findings are particularly encouraging in the light of the known role of self-efficacy in relation to the management of chronic pain [41]. In addition, a high percentage of participants reported that they had learnt to improve the way they lived and cared for themselves as a result of the lessons and this percentage increased between 6 and 12 months from 81% to 87%, reflecting the reinforcing self-learning nature of the Alexander Technique and continuous benefit of applying it. This result is in line with the main clinical findings of the trial, which showed that the reduction in NPQ score observed at 6 months was maintained to 12 months, indicating little or no loss of benefit once the Alexander lessons had ceased [16]. Longer-term retention of skills learnt in Alexander lessons has also been reported previously [42].

Interestingly, there was no association between participants' self-efficacy at baseline and clinical outcome (NPQ), suggesting that good outcomes were not dependent on a pre-existing strong belief in ability to reduce pain. Rather, participants became more confident in their ability to reduce their pain as a result of attending Alexander lessons and learning the Technique, and this is what led to better clinical outcomes. The lack of baseline predictors of clinical outcome, taken together with the broad trial inclusion criteria that ensured a diverse and representative non-specific chronic neck pain population, suggest that Alexander lessons may be widely appropriate, with no differential effects identified for different subgroups of patients. Research in other areas has also shown that certain education-based interventions can lead to increased self-efficacy, with associated improvement in clinical outcomes, including pain reduction [43].

The significant change in exercise that was reported in the Alexander group is interesting, particularly as specific exercise advice does not form part of Alexander teaching. If it signifies that the ATLAS Alexander group participants increased the amount of general exercise or activity they engaged in, this would be consistent with the reported increase in self-efficacy and decrease in NPQ score. It would also suggest a potential explanation for the finding in the ATEAM back pain trial that those participants in the group offered 24 Alexander lessons who also received a prescription for general exercise were found to gain no additional benefit from the exercise prescription [18]. Perhaps most of the participants in that group were already engaging in more activity?

One surprising finding in the current analysis is the lack of significant change in the Perceived Stress Scale when explored as the outcome of interest. Other than changes to diet, this is the only outcome that did not change significantly following Alexander lessons. Anecdotally, stress reduction is one of the main reported benefits of Alexander lessons, albeit that stress/anxiety is not currently reported as a main reason for beginning lessons [17]. Moreover, qualitative research, sometimes embedded in controlled trials, has reported improvement in psychological well-being and reduced performance-related anxiety following Alexander lessons [[20], [27], [28]]. A potential explanation for the lack of significant change in the Perceived Stress Scale may relate to our use of the short-form version (maximum possible score: 16), consisting of 4 items rather than the standard 10 or 14. Baseline scores in the ATLAS trial were approximately 6, which is typical for a UK general population [44]. This unexpectedly low baseline score left limited scope for significant reduction during the trial. Further research is warranted in this area using the more sensitive 10- or 14-item scale, together with a range of other outcome measures for stress and anxiety.

Future research could usefully evaluate the relative benefit of one-to-one Alexander lessons alone and one-to-one lessons combined with some group classes to explore possible differences in relative teaching/learning efficiency and cost/benefit ratios. Furthermore it would be worthwhile to evaluate the effectiveness of an initial course of acupuncture for rapid pain relief in the chronic neck pain population, followed by Alexander lessons for long-term self-efficacy and self-care.

## 5 Conclusions

Alexander lessons promote self-efficacy and self-care by imparting knowledge and skills that help people improve the way they live and care for themselves, leading to long-term reduction in chronic neck pain.

## Funding

The ATLAS trial was funded by a clinical studies grant from Arthritis Research UK (grant number 19702). The funding body had no role in this analysis, the writing of the article or the decision to submit for publication.

## Conflicts of interest

The authors have no conflicts of interest.
