# Supplementary material for: Self-efficacy and self-care-related outcomes following Alexander Technique lessons for people with chronic neck pain in the ATLAS randomised, controlled trial
Source: Eur J Integr Med. 2018 Jan;17:64–71. doi: 10.1016/j.eujim.2017.11.006 (PMC5842361; doi:10.1016/j.eujim.2017.11.006)
Supplement: Supplementary file 1 [file mmc1.docx]

**Supplementary Table 1:**  **Baseline factors assessed as potential predictors or moderators of the Northwick Park Neck Pain and Disability Questionnaire score at 12 months**

|  | **Coefficient** | **95% CI** | | **p-value** | **Moderator interaction p-value** |
| --- | --- | --- | --- | --- | --- |
|  |  | **Lower** | **Upper** |  |  |
| Age | -0.01 | -0.15 | 0.12 | 0.87 | 0.51 |
| Gender: female vs. male | 1.41 | -1.71 | 4.53 | 0.36 | 0.54 |
| Ethnicity: White vs. non-white | 0.78 | -5.70 | 7.27 | 0.81 | 0.14 |
| Age when left full time education | 0.01 | -0.14 | 0.15 | 0.92 | 0.76 |
| Duration of neck pain | 0.00 | -0.02 | 0.02 | 0.97 | 0.23 |
| Neck pain worse:  with stress  when tired | -0.96  2.60 | -4.04  -0.76 | 2.13  5.97 | 0.53  0.12 | 0.42  0.31 |
| Chronic Pain Self-Efficacy Scale | -0.69 | -1.98 | 0.61 | 0.29 | 0.34 |
| Perceived Stress Scale | 0.27 | -0.17 | 0.71 | 0.22 | 0.23 |
| In paid employment | -2.60 | -6.00 | 0.80 | 0.13 | 0.13 |

Supplementary Table 2: Differences between outcomes following Alexander Technique lessons and acupuncture that support conclusions regarding the influence of specific factors unique to each intervention^†^. Analyses are descriptive only, the trial was not powered for a comparison of Alexander lessons and acupuncture

| **Outcome** | **Difference from usual care alone at 6 months** | | | | **Difference from usual care alone at 12 months** | | | |
| --- | --- | --- | --- | --- | --- | --- | --- | --- |
|  | **Alexander Technique lessons (95% CI)** | **p-value** | **Acupuncture (95% CI)** | **p-value** | **Alexander Technique lessons (95% CI)** | **p-value** | **Acupuncture (95% CI)** | **p-value** |
| Chronic Pain Self-efficacy Scale | 1.09 (0.63 to 1.55) | <0.001 | 0.82 (-0.49 to 1.16) | <0.001 | 0.81 (0.37 to 1.24) | 0.001 | 0.65 (0.18 to 1.13) | 0.009 |
| Perceived Stress Scale | -0.02 (-0.84 to 0.80) | 0.97 | 0.14 (-0.58 to 0.87) | 0.69 | -0.12 (-0.79 to 0.54) | 0.70 | 0.11 (-0.53 to 0.75) | 0.74 |
| 'Can you use/apply things you learned from the care in everyday life situations, to reduce pain?' | 1.30 (0.94 to 1.66) | <0.001 | 0.29 (-0.01 to 0.60) | 0.06 | 1.11 (0.83 to 1.38) | <0.001 | 0.23 (-0.06 to 0.52) | 0.12 |
| 'To what extent are you able to put into practice the advice or teaching you received?' | 2.20 (0.91 to 3.50) | 0.002 | 1.52 (-0.48 to 2.55) | 0.005 | 1.01 (-0.20 to 2.21) | 0.10 | 0.30 (-0.95 to 1.55) | 0.63 |
| 'To what extent are the changes you have been making helpful to you?' | 2.23 (0.96 to 3.50) | 0.001 | 1.52 (0.53 to 2.50) | 0.004 | 1.21 (0.04 to 2.38) | 0.04 | 0.82 (-0.49 to 2.12) | 0.21 |
| **Yes/No response questions** | **Odds ratio (95% CI)** | **p-value** | **Odds ratio (95% CI)** | **p-value** | **Odds ratio (95% CI)** | **p-value** | **Odds ratio (95% CI)** | **p-value** |
| 'Did you learn to improve the way you live and care for yourself?' | 15.32 (8.79 to 26.68) | <0.001 | 5.35 (3.47 to 8.23) | <0.001 | 23.98 (10.02 to 57.41) | <0.001 | 5.39 (3.32 to 8.74) | <0.001 |
| 'Did you make any changes related to': |  |  |  |  |  |  |  |  |
| Diet | 1.82 (0.60 to 5.47) | 0.29 | 5.32 (2.29 to 12.32) | <0.001 | 1.69 (0.88 to 3.25) | 0.12 | 2.74 (1.45 to 5.21) | 0.002 |
| Exercise | 2.12 (1.12 to 3.98) | 0.02 | 2.10 (1.17 to 3.79) | 0.01 | 3.51 (1.99 to 6.21) | <0.001 | 2.62 (1.57 to 4.38) | <0.001 |
| Relaxation | 19.76 (10.37 to 37.63) | <0.001 | 8.14 (4.13 to 16.05) | <0.001 | 17.45 (8.92 to 34.14) | <0.001 | 6.06 (3.86 to 9.52) | <0.001 |
| Rest | 20.31 (9.15 to 45.06) | <0.001 | 7.22 (3.31 to 15.78) | <0.001 | 8.33 (4.64 to 14.99) | <0.001 | 2.85 (1.63 to 5.01) | <0.001 |
| Work | 20.95 (8.68 to 50.54) | <0.001 | 8.19 (3.75 to 17.86) | <0.001 | 6.98 (3.19 to 15.24) | <0.001 | 2.22 (1.03 to 4.75) | 0.04 |

^†^Rather than resulting from increased contact time and non-specific effects of attention and touch; Adjustments were made for baseline NPQ score, duration of neck pain, age, gender and city as a fixed effect and GP practice as a random effect using robust standard errors*;* The detailed findings for the acupuncture intervention are reported elsewhere [30]; CI: confidence interval.

**Supplementary Table 3: Summaries of Alexander Technique principles and activities engaged in during the lessons***

|  | **n (%) of participants who were taught the principle/activity at least once during their lessons**  **N=150** | **Frequency of principles taught / activities used during lessons**^‡^  **Median (min, max)**  **N=150** |
| --- | --- | --- |
| **Alexander Technique principles^†^** |  |  |
| 'Nature of habit' | 144 (96.0) | 0.30 (0,1.00) |
| 'Inhibition / withholding consent / non-doing' | 140 (93.3) | 0.50 (0, 1.00) |
| 'Direction / giving consent' | 140 (93.3) | 0.65 (0, 1.00) |
| 'Primary control' | 135 (90.0) | 0.35 (0, 1.00) |
| 'Use and functioning' | 119 (79.3) | 0.20 (0, 1.00) |
| 'Conscious guidance and control' | 119 (79.3) | 0.25 (0, 1.00) |
| 'Endgaining / means whereby' | 118 (78.7) | 0.20 (0, 1.00) |
| 'Psycho-physical unity' | 115 (76.7) | 0.22 (0, 1.00) |
| 'Sensory appreciation' | 111 (74.0) | 0.15 (0, 1.00) |
| 'The right thing does itself' | 107 (71.3) | 0.15 (0, 0.95) |
| **Activities used in lessons to practice applying the Technique / AT skills** |  |  |
| 'Table work' | 148 (98.7) | 0.95 (0, 1.00) |
| 'Chair work' | 146 (97.3) | 0.95 (0, 1.00) |
| 'Semi-supine as home practice' | 141 (94.0) | 0.25 (0, 1.00) |
| 'Attending to support (from ground / chair etc)' | 131 (87.3) | 0.30 (0, 1.00) |
| 'Being still / coming to quiet' | 123 (82.0) | 0.51 (0, 1.00) |
| 'Monkey' | 123 (82.0) | 0.30 (0, 0.86) |
| 'Breathing / whispered ah’ | 118 (78.7) | 0.21 (0, 1.00) |
| 'Developing a practical understanding of AO and AA joints and of hip, knee & ankle joints, and the co-ordination of their movements' | 116 (77.3) | 0.15 (0, 1.00) |
| 'Eyes leading movement' | 116 (77.3) | 0.20 (0, 1.00) |
| 'Walking' | 104 (69.3) | 0.10 (0, 0.70) |
| 'Developing integrated field of attention and awareness' | 96 (64.0) | 0.13 (0, 1.00) |
| 'Keeping eyes lively and developing panoramic vision' | 96 (64.0) | 0.10 (0, 1.00) |
| 'Use of the AT to avoid pain anticipation & consequent pain-producing behaviour' | 92 (61.3) | 0.12 (0, 1.00) |
| 'Other activity' | 86 (57.3) | 0.10 (0, 1.00) |
| 'Reaching, picking things up or supporting a weight' | 70 (46.7) | 0.00 (0, 0.65) |
| 'Co-ordinating arm and back muscle activity' | 45 (30.0) | 0.00 (0, 0.75) |
| 'Climbing / descending stairs' | 43 (28.7) | 0.00 (0, 0.15) |
| 'Appreciation and use of the spine as one thing’ | 42 (28.0) | 0.00 (0, 0.75) |
| 'Inhibition of irrelevant movements' | 41 (27.3) | 0.00 (0, 0.80) |
| 'Co-ordinating leg and back muscle activity' | 34 (22.7) | 0.00 (0, 0.35) |
| 'Hands on back of chair' | 29 (19.3) | 0.00 (0, 0.46) |
| ‘Thinking not doing applied to any activity' | 29 (19.3) | 0.00 (0, 0.45) |
| 'Working at a computer / writing' | 19 (12.7) | 0.00 (0, 0.20) |
| 'Speaking / vocalising' | 14 (9.3) | 0.00 (0, 0.25) |

*For each lesson, teachers were asked to indicate which Alexander principles were particularly focused on, and which activities and procedures were taught / used to engage the participant in; **^†^**The principles listed in the log book were based on the National Occupational Standards for Alexander teaching [33]; ^‡^Values close to 1 represent principles / activities that were a main focus of almost every lesson; The 21 participants who did not attend any Alexander lessons did not have a logbook so are excluded from the analyses and there was one person who did not provide any information on lesson content.
